# Supplementary material for: Antenatal and perinatal service delivery associations with breastfeeding outcomes in Nepal: Analysis of the 2016 Nepal Demographic and Health Survey
Source: PLOS Glob Public Health. 2023 Apr 17;3(4):e0001824. doi: 10.1371/journal.pgph.0001824 (PMC10109470; doi:10.1371/journal.pgph.0001824)
Supplement: S1 Table — (DOCX) [file pgph.0001824.s001.docx]

| Supplementary Table 1. Distribution of age-appropriate infant and young child feeding (IYCF) practice according to infant age group. | | | | | | | | |
| --- | --- | --- | --- | --- | --- | --- | --- | --- |
|  |  | **Total**  **N (%)^a^** | **Overall age-appropriate feeding practice**  **(0-23 months)**  **N (%)^b^** | **P-value^c^** | **Exclusive breastfeeding**  **(0-5 months)**  **N (%)^b^** | **P-value^c^** | **Continued breastfeeding with complementary foods**  **(6-23 months)**  **N (%)^b^** | **P-value^c^** |
| **Total children (0-23 months)** | | 1938 (100) | 1665 (85.9) |  | N/A |  | N/A |  |
| **IYCF age group** | |  |  | **<0.0005** |  |  |  |  |
|  | 0-5 months | 443 (22.9) | 293 (66.1) |  | 293 (66.1) |  | N/A |  |
|  | 6-23 months | 1495 (77.1) | 1372 (91.8) |  | N/A |  | 1372 (91.8) |  |
| **Child factors** | |  |  |  |  | | | |
| Sex^d^ |  |  |  | 0.978 |  | 0.671 |  | 0.563 |
|  | Male | 1043 (53.8) | 895 (85.9) |  | 157 (67.1) |  | 738 (91.3) |  |
|  | Female | 895 (46.2) | 769 (85.9) |  | 136 (65.1) |  | 633 (92.3) |  |
| Current Age (months) | |  |  | **<0.0005** |  | **<0.001** |  | **<0.001** |
|  | 0-1 | 159 (8.2) | 127 (79.4) |  | 127 (79.4) |  | N/A |  |
|  | 2-3 | 160 (8.2) | 115 (72.3) |  | 115 (72.3) |  | N/A |  |
|  | 4-5 | 124 (6.4) | 51 (40.8) |  | 51 (40.8) |  | N/A |  |
|  | 6-8 | 235 (12.1) | 196 (83.4) |  | N/A |  | 196 (83.4) |  |
|  | 9-11 | 264 (13.6) | 250 (95.1) |  | N/A |  | 250 (95.1) |  |
|  | 12-17 | 504 (26.0) | 482 (95.6) |  | N/A |  | 482 (95.6) |  |
|  | 18-23 | 492 (25.4) | 444 (90.2) |  | N/A |  | 444 (90.2) |  |
| Size at birth^d^ | |  |  | 0.627 |  | 0.639 |  | 0.290 |
|  | Very small/small | 331 (17.1) | 290 (87.6) |  | 41 (60.3) |  | 249 (94.3) |  |
|  | Average | 1318 (68.0) | 1124 (85.3) |  | 211 (67.6) |  | 913 (90.8) |  |
|  | Large | 288 (14.9) | 250 (86.8) |  | 41 (65.1) |  | 209 (92.9) |  |
| Birth interval | |  |  | 0.158 |  | 0.136 |  | 0.122 |
|  | First birth episode | 792 (40.9) | 684 (86.4) |  | 119 (67.2) |  | 565 (91.9) |  |
|  | 18 months or less | 100 (5.2) | 79 (79.0) |  | 6 (35.3) |  | 72 (86.7) |  |
|  | 19-36months | 428 (22.1) | 360 (84.1) |  | 61 (65.6) |  | 299 (89.3) |  |
|  | 37months+ | 617 (32.0) | 542 (87.7) |  | 106 (67.9) |  | 435 (94.4) |  |
| **Maternal factors** | | |  |  |  | | | |
| Current age (years)^d^ | |  |  | **0.016** |  | 0.952 |  | 0.185 |
|  | 15-19 | 284 (14.7) | 229 (80.6) |  | 61 (65.6) |  | 168 (88.0) |  |
|  | 20-29 | 1311 (67.7) | 1136 (87.0) |  | 190 (66.7) |  | 946 (92.9) |  |
|  | 30-39 | 314 (16.2) | 271 (86.3) |  | 40 (63.5) |  | 230 (92.0) |  |
|  | 40-49 | 29 (1.5) | 28 (100)^b^ |  | 1 (100) |  | 27 (100) |  |
| Occupation |  |  |  | **0.001** |  | 0.161 |  | **<0.001** |
|  | Did not work/ household duties | 913 (47.1) | 749 (82.0) |  | 153 (64.6) |  | 596 (88.2) |  |
|  | Agricultural work (paid and unpaid) | 803 (41.4) | 719 (89.4) |  | 116 (71.2) |  | 602 (94.1) |  |
|  | Non-agricultural work (paid) | 221 (11.4) | 197 (89.1) |  | 23 (54.8) |  | 174 (97.2) |  |
| Highest education level^d^ | |  |  | 0.901 |  | 0.242 |  | **0.016** |
|  | No education | 555 (28.6) | 473 (85.2) |  | 74 (71.2) |  | 399 (88.3) |  |
|  | Primary | 380 (19.7) | 324 (85.3) |  | 58 (67.4) |  | 266 (90.5) |  |
|  | Secondary | 707 (36.5) | 612 (86.6) |  | 104 (60.1) |  | 509 (95.1) |  |
|  | Higher education (tertiary +) | 295 (15.2) | 255 (86.4) |  | 57 (70.4) |  | 198 (92.5) |  |
| **Antenatal and perinatal service delivery** | | |  |  |  |  |  |  |
| Number of antenatal visits | |  |  | 0.291 |  | 0.231 |  | 0.197 |
|  | 0-3 visits | 552 (28.5) | 470 (85.1) |  | 76 (67.9) |  | 394 (89.5) |  |
|  | 4 visits | 580 (30.0) | 513 (88.4) |  | 94 (70.7) |  | 420 (94.0) |  |
|  | 5-7 visits | 634 (32.7) | 540 (85.2) |  | 98 (65.8) |  | 442 (91.1) |  |
|  | 8+ visits | 172 (8.9) | 141 (82.0) |  | 25 (51.0) |  | 116 (94.3) |  |
| Type of delivery | |  |  | 0.854 |  | 0.521 |  | 0.300 |
|  | Vaginal | 1742(89.9) | 1496 (85.9) |  | 265 (66.6) |  | 1231(91.5) |  |
|  | Caesarean | 195 (10.1) | 169 (86.2) |  | 28 (60.9) |  | 141 (94.0) |  |
| Place of delivery | |  |  | **0.084** |  | 0.874 |  | **0.003** |
|  | Home | 666 (34.4) | 567 (85.1) |  | 103 (67.8) |  | 464 (90.3) |  |
|  | Health facility | 1178 (60.8) | 1025 (87.0) |  | 177 (65.1) |  | 848 (93.6) |  |
|  | Other | 94 (4.8) | 73 (77.7) |  | 12 (63.2) |  | 60 (80.0) |  |
| Child put on mother’s bare skin after birth^e^ | |  |  | 0.521 |  | 0.578 |  | 0.991 |
|  | No | 695 (35.9) | 592 (85.2) |  | 106 (63.9) |  | 485 (91.9) |  |
|  | Yes | 1222 (63.0) | 1054 (86.3) |  | 183 (67.0) |  | 871 (91.9) |  |
|  | Don’t know^g^ |  |  |  |  |  |  |  |
| Breastfeeding counselling from any healthcare provider during first 2 days | |  |  | 0.339 |  | 0.711 |  | 0.385 |
|  | No | 882 (45.5) | 751 (85.1) |  | 134 (65.7) |  | 617 (91.0) |  |
|  | Yes | 1049 (54.1) | 912 (86.9) |  | 159 (67.7) |  | 753 (92.5) |  |
| Observation of breastfeeding by any healthcare provider during first 2 days | |  |  | 0.344 |  | 0.531 |  | 0.639 |
|  | No | 959 (49.5) | 816 (85.1) |  | 147 (64.8) |  | 701 (92.1) |  |
|  | Yes | 974 (50.3) | 847 (87.0) |  | 145 (68.1) |  | 669 (91.4) |  |
| **Household Level Factors** | | |  |  |  | | | |
| Wealth quintile | |  |  | 0.218 |  | 0.235 |  | **0.094** |
|  | Poorest | 405 (20.9) | 364 (89.7) |  | 73 (70.9) |  | 290 (96.0) |  |
|  | Poorer | 408 (21.0) | 352 (86.3) |  | 51 (62.2) |  | 301 (92.3) |  |
|  | Middle | 444 (22.9) | 379 (85.4) |  | 75 (73.5) |  | 304 (89.1) |  |
|  | Richer | 398 (20.6) | 334 (83.9) |  | 48 (60.0) |  | 285 (89.9) |  |
|  | Richest | 283 (14.6) | 237 (83.7) |  | 45 (59.2) |  | 191 (92.3) |  |
| Husband’s highest education level^f^ | |  |  | 0.794 |  | 0.743 |  | 0.849 |
|  | No education | 255 (13.1) | 220 (86.6) |  | 39 (70.9) |  | 182 (91.0) |  |
|  | Primary | 435 (22.5) | 368 (84.6) |  | 59 (62.1) |  | 309 (90.6) |  |
|  | Secondary | 863 (44.6) | 748 (86.6) |  | 134 (67.3) |  | 614 (92.3) |  |
|  | Higher education (tertiary +) | 377 (19.5) | 322 (85.2) |  | 62(64.6) |  | 260 (92.2) |  |
| Ethnicity |  |  |  | **<0.001** |  | 0.841 |  | **<0.001** |
|  | Brahman/Chhetri | 524 (27.0) | 464 (88.7) |  | 100 (69.0) |  | 364 (96.0) |  |
|  | Other Terai Castes | 387 (20.0) | 316 (81.4) |  | 53 (63.9) |  | 262 (86.2) |  |
|  | Dalits | 268 (13.8) | 223 (83.2) |  | 34 (58.6) |  | 189 (90.0) |  |
|  | Newar | 61 (3.1) | 58 (95.1) |  | 5 (71.4) |  | 53 (98.1) |  |
|  | Janajati | 546 (28.2) | 487 (89.2) |  | 74 (67.3) |  | 413 (94.7) |  |
|  | Muslim and other | 151 (7.8) | 117 (77.0) |  | 26 (65.0) |  | 91 (81.3) |  |
| Food Security | |  |  | 0.493 |  | 0.916 |  | 0.504 |
|  | Secure | 791 (40.8) | 674 (85.2) |  | 121 (65.8) |  | 552 (91.1) |  |
|  | Not secure | 1147 (59.2) | 991 (86.4) |  | 171 (66.3) |  | 819 (92.2) |  |
|  |  |  |  |  |  |  |  |  |
| **Community Level Factors** | |  |  |  |  | | | |
| Ecological zone^d^ | |  |  | **0.007** |  | 0.814 |  | **<0.001** |
|  | Terai | 1065 (55.0) | 887 (83.3) |  | 161 (66.0) |  | 726 (88.4) |  |
|  | Mountain | 127 (6.5) | 112 (88.2) |  | 21 (72.4) |  | 91 (93.8) |  |
|  | Hill | 746 (38.5) | 665 (89.3) |  | 111 (65.3) |  | 555 (96.4) |  |
| Province |  |  |  | **<0.001** |  | **0.020** |  | **<0.001** |
|  | Province 1 | 335 (17.3) | 285 (84.8) |  | 37 (55.2) |  | 248 (92.2) |  |
|  | Province 2 | 501 (25.8) | 394 (78.6) |  | 72 (60.0) |  | 322 (84.5) |  |
|  | Province 3 | 305 (15.7) | 266 (87.2) |  | 37 (57.8) |  | 228 (95.0) |  |
|  | Province 4 | 162 (8.4) | 147 (90.7) |  | 27 (73.0) |  | 120 (96.0) |  |
|  | Province 5 | 354 (18.3) | 318 (89.6) |  | 61 (75.3) |  | 257 (93.8) |  |
|  | Province 6 | 118 (6.1) | 104 (88.1) |  | 23 (71.9) |  | 81 (94.2) |  |
|  | Province 7 | 162 (8.4) | 151 (93.2) |  | 36 (83.7) |  | 115 (96.6) |  |
| Type of place of residence | |  |  | 0.852 |  | 0.237 |  | 0.348 |
|  | Urban | 1042 (53.8) | 894 (85.7) |  | 152 (63.3) |  | 742 (92.5) |  |
|  | Rural | 895 (46.2) | 771 (86.0) |  | 141 (69.5) |  | 630 (91.0) |  |
|  |  |  |  |  |  |  |  |  |

Notes:

Bold text indicates P-value <0.1 and included in subsequent regression models.

N/A Not applicable.

^a^ Column percentage.

^b^ Row percentage.

^c^ P-value from complex sample chi-squared tests.

^d^ One missing value excluded from analysis.

^e^ Two missing values excluded from analysis.

^f^ Five missing value excluded from analysis.

^g^ ‘Don’t know’ values excluded from further analysis.
