# Supplementary material for: Antenatal and perinatal service delivery associations with breastfeeding outcomes in Nepal: Analysis of the 2016 Nepal Demographic and Health Survey
Source: PLOS Glob Public Health. 2023 Apr 17;3(4):e0001824. doi: 10.1371/journal.pgph.0001824 (PMC10109470; doi:10.1371/journal.pgph.0001824)
Supplement: S2 Table — (DOCX) [file pgph.0001824.s002.docx]

| Supplementary Table 2. Adjusted odds ratios for age-appropriate infant and young child feeding (IYCF) practice according to infant age group. | | | |
| --- | --- | --- | --- |
|  | **Overall age-appropriate feeding practice**  **(0-23 months)**  **N=1938**  **Adjusted OR (95% CI)** | **Exclusive breastfeeding**  **(0-5 months)**  **N=443**  **Adjusted OR (95% CI)** | **Continued breastfeeding with complementary foods**  **(6-23 months)**  **N=1495**  **Adjusted OR (95% CI)** |
| **Sex of child** |  |  |  |
| Male | 1.00 *Ref* | 1.00 *Ref* | 1.00 *Ref* |
| Female | 0.99 (0.68-1.44) | 0.844 (0.52-1.36) | 1.12 (0.68-1.85) |
| **Age of child (months)** |  |  |  |
| 0-1 | 1.00 *Ref* | 1.00 *Ref* |  |
| 2-3 | 0.68 (0.35-1.32) | 0.68 (0.35-1.31) |  |
| 4-5 | **0.16 (0.08-0.30) ***** | **0.16 (0.09-0.30) ***** |  |
| 6-8 | 1.31 (0.72-2.39) |  | **0.20 (0.09-0.44) ***** |
| 9-11 | **5.09 (2.25-11. 50)***** |  | 0.90 (0.38-2.09) |
| 12-17 | **5.60 (2.73-11.47) ***** |  | 1.00 *Ref* |
| 18-23 | **2.46 (1.31-4.64) **** |  | **0.41 (0.20-0.85) *** |
| **Age of mother (years)^a^** |  |  |  |
| 15-19 | 0.85(0.52-1.40) |  |  |
| 20-29 | 1.00 *Ref* |  |  |
| 30-39 | 0.90 (0.56-1.43) |  |  |
| 40-49 | **7.35 (1.14-47.29) *** |  |  |
| **Mother’s highest education level** |  |  |  |
| No education |  |  | 1.00 *Ref* |
| Primary |  |  | 0.98 (0.55-1.77) |
| Secondary |  |  | 1.72 (0.91-3.25) |
| Higher education |  |  | 0.76 (0.34-1.70) |
| **Mother’s occupation** |  |  |  |
| Did not work/ household duties | 1.00 *Ref* |  | 1.00 *Ref* |
| Agricultural work (paid and unpaid) | 1.27 (0.83-1.95) |  | 1.39 (0.76-2.54) |
| Non-agricultural work (paid) | 1.15 (0.62-2.13) |  | **3.67 (1.04-13.02) *** |
| **Place of delivery** |  |  |  |
| Home | 1.00 *Ref* |  | 1.00 *Ref* |
| Health facility | 1.10 (0.75-1.62) |  | 1.55 (0.92-2.63) |
| Other | 0.66 (0.29-1.53) |  | 0.56 (0.22-1.45) |
| **Ethnicity** |  |  |  |
| Brahman/Chhetri | 1.00 *Ref* |  | 1.00 *Ref* |
| Other Terai Castes | 0.75 (0.44-1.29) |  | 0.52 (0.23-1.19) |
| Dalits | 0.60 (0.35-1.02) |  | 0.43 (0.18-1.05) |
| Newar | 2.39 (0.61-9.42) |  | 1.58 (0.14-17.25) |
| Janajati | 1.07 (0.66-1.74) |  | 0.73 (0.31-1.72) |
| Muslim and other | 0.54 (0.29-1.02) |  | **0.37 (0.15-0.87) *** |
| **Ecological zone^a^** |  |  |  |
| Terai | 0.86 (0.56-1.30) |  | 0.55 (0.23-1.30) |
| Mountain | 0.82(0.42-1.60) |  | 0.56 (0.25-1.25) |
| Hill | 1.00 *Ref* |  | 1.00 *Ref* |
| **Province** |  |  |  |
| Province 1 | 0.96 (0.53-1.76) | 0.76 (0.31-1.86) | 1.28 (0.46-3.53) |
| Province 2 | 0.98 (0.50-1.91) | 1.07 (0.43-2.64) | 1.19 (0.41-3.46) |
| Province 3 | 1.00 Ref | 1.00 *Ref* | 1.00 *Ref* |
| Province 4 | 1.65 (0.84-3.25) | 1.80 (0.74-4.38) | 1.31 (0.37-4.61) |
| Province 5 | **1.93** **(1.02-3.67) *** | 2.36 (1.00-5.58) | 1.87 (0.63-5.59) |
| Province 6 | 1.69 (0.81-3.51) | 2.29 (0.92-5.72) | 0.78 (0.22-2.75) |
| Province 7 | **3.11** **(1.54-6.30) **** | **4.33 (1.64-11.42) **** | 2.08 (0.58-7.39) |
|  |  |  |  |
| **Wealth quintile** |  |  |  |
| Poorest |  |  | 1.87 (0.81-4.31) |
| Poorer |  |  | 0.92 (0.44-1.93) |
| Middle |  |  | 1.00 *Ref* |
| Richer |  |  | 0.81 (0.39-1.64) |
| Richest |  |  | 1.00 (0.41-2.43) |
| **Factors included in model** | Sex of child, age of child (months), age of mother (years), mother’s occupation, place of delivery, ethnicity, ecological zone, province | Sex of child, age of child (months), province | Sex of child, age of child (months), mother’s highest education level, mother’s occupation, place of delivery, ethnicity, ecological zone, province, wealth quintile |

Notes:

Bold text indicates statistically significant result.

*P <0.05, ** P <0.01, *** P<0.001.

^a^ 1 missing value that was excluded from analysis.
