# Supplementary material for: Antenatal and perinatal service delivery associations with breastfeeding outcomes in Nepal: Analysis of the 2016 Nepal Demographic and Health Survey
Source: PLOS Glob Public Health. 2023 Apr 17;3(4):e0001824. doi: 10.1371/journal.pgph.0001824 (PMC10109470; doi:10.1371/journal.pgph.0001824)
Supplement: Attachment — Submitted filename: Response to reviewers.docx [file pgph.0001824.s003.docx]

**Response to Reviewers’ Comments**
==============================
We invite you to submit a revised version of your manuscript, taking into consideration the comments received from the three reviewers. In particular, we agree with the comment of reviewer #1 regarding the need to clarify the indicator related to observed feeding and counselling, and highlight this as a limitation. Please provide a rationale for why you decided to treat antenatal care as a dichotomous variable. Finally, please consider disaggregating the variable, age-appropriate breastfeeding practice, by age category.
==============================
These points have all been addressed below.

Reviewer #1: 

Major comments
Overall the paper would benefit from some clarity in the indicators. In particular, the indicator related to observed feeding and counselling. This is often referred to in a confusing way, especially when in relation to skin to skin contact and early initiation of breastfeeding. The indicator is defined as being measured over 2 days compared to the first hour after birth.

The definitions have been clarified in the Methods.

Explanatory variables (lines 196-200).

“…infant-mother skin-to-skin contact post-delivery (child put directly on the bare skin of the mother’s bare chest immediately after the birth) (yes; no), breastfeeding counselling (yes; no) by any healthcare provider within the first two days post-delivery, and breastfeeding observation (yes; no) by any health healthcare provider within the first two days post-delivery.”

Outcome variables (lines 167-168)

“… early initiation of breastfeeding, defined by WHO as ‘breastfeeding within the first hour of life’”.

We have now used these terms consistently throughout the document to avoid confusion.

The association is therefore difficult to interpret unless we assume that the observation took place within the first hour after birth. Please do consider making clearer throughout the text and also highlighting it as a limitation.

In the discussion (lines 395-399) we explain:

“Although the temporality of the relationship between observation of breastfeeding by a healthcare provider in the first two days after delivery and breastfeeding within the first hour cannot be discerned here, it is plausible that breastfeeding within one hour of delivery may be facilitated when health professionals are available for observation and troubleshooting post-delivery.”

We also highlight in the limitations (lines 474-479):

“Other limitations relate to measurement bias. “Observation of breastfeeding within the first two days post-delivery” and “counselling of breastfeeding within the first two days post-delivery” cannot be shown to have preceded the event of “breastfeeding within the first hour”. These variables were measured in a dichotomous “yes/no” format and the exact timings of observation or counselling were not recorded.”

I also find the order in which results are presented hard to follow. I think presenting skin to skin, then early initiation, then age-appropriate feeding practices is a more logical order and would improve the flow of the paper.

We have rearranged the Results, Discussion and data in the Tables so that early initiation (breastfeeding within the first hour) is followed by age-appropriate breastfeeding practices. Infant-mother skin-to-skin contact post-delivery is an explanatory variable, not an outcome variable, and is therefore discussed in the context of the outcome variables.

Minor comments
Abstract – I don’t think the abstract concisely captures the main points of the paper. Some reworking would benefit the paper and make it more inviting to readers

We have made minor edits to the Abstract body and reworded the conclusion to focus on significance for practice (lines 64-73).

“Further promotion of infant-mother skin-to-skin contact post-delivery (including after caesarean delivery) and the observation of early breastfeeding may increase the rate of breastfeeding within one hour of delivery. This service improvement can be achieved through enhanced implementation of the Baby Friendly Hospitals Initiative and the provision of effective training and sufficient practice for skilled birth attendants. Additional postnatal support should be considered for mothers with infants approaching 4-5 months to improve the low rate of exclusive breastfeeding observed in this age-group.”

Table 1 - Please define weights for size at birth

In the limitations (lines 479-481) we have indicated that size of infant at birth was self-reported and is a source of measurement bias. No birthweight data is available in the DHS survey.

“Also, the size at birth of the infant was self-reported by the mother as small/very small, average, or large, which is inaccurate and subjective.”

Line 201 – the term recent is confusing in this sentence

Deleted the term recent.

Lines 220-223 – please could you elaborate on the meaning of this a little more

The response to the first question of reviewer #3 clarifies the methodology used for collinear variables in the model.

In the results section the wording of these lines has been clarified (lines 277-280):

“Since counselling regarding breastfeeding within two days of delivery and observation of breastfeeding within two days of delivery were highly associated with each other (p<0.0005), only one of these variables, observation of breastfeeding within two days of delivery, was included in the multivariable model.”

Page 17 (sorry, line numbers disappeared) – I think the discussion on supply could benefit from further information. Are there physiological reasons for diminished supply or is it due to mixed feeding. Do other studies exist or is this a research gap in this context? Is it mothers perception of supply? Please define how this is determined.

We have added the following sentence in the Discussion (lines 424-426) along with an additional reference.

“Physiological reductions in serum prolactin levels with time post-delivery and reduction in supply with mixed feeding have been associated with reduced maternal breastmilk supply (27).”

Page 18 – 3rd paragraph – please pull apart and comment more clearly on what is needed in terms of early breastfeeding advice. For example, is it the responsibility of skilled birth attendants to promote immediate skin to skin and the support the mother within the first hour following birth with attachment and feeding? Also how can this be improved for women with non vaginal deliveries? Would promotion of skin to skin and early attachment among obstetricians performing caesareans help? Similarly, with those who do not have skilled attendance. Are there local or regional examples of good practice and impact that can be cited?

We have included some more information on the Baby Friendly Hospitals Initiative and Skilled Birth Attendants in Nepal in the Introduction (lines 125-128) and Discussion (lines 414-418) and described that these policies align with WHO-endorsed breastfeeding recommendations. In the Conclusion (lines 453-501) and Abstract (lines 66-68) we focus on strengthening the implementation of these policies to achieve better outcomes for the early initiation of breastfeeding. To provide more detail than this is beyond the scope of the paper.

Reviewer #2: No comments for response.

Reviewer #3: 
Lines 165-167: The sentence beginning with “Where two explanatory variables” needs further explanation. It’s not clear exactly what this means but if whether the reason to select one variable is due to multicollinearity concerns, I would recommend looking at the VIF for each variable.

This paragraph is concerned with managing multicollinearity of categorical explanatory variables (predictors) in the logistic regression model. When concerned about potential multicollinearity due to unstable coefficient estimates, the relationships between variables were explored. The most direct way to determine the extent to which two categorical variables are associated with each other is to cross-tabulate them and test for association using the Chi-square test. Omitting one of the correlated variables, if practical, can reduce the multicollinearity to a great extent.

We have clarified the wording in the Methods (lines 233-237):

“In multivariable model testing potential collinearity of explanatory variables was explored using Chi-square tests when the coefficient estimates were very unstable. Where two variables were highly associated with each other, one of the variables was removed from the multivariable logistic regression model.”

I have a couple of concerns about the “age-appropriate breastfeeding practice” outcome variable. I can see how on its surface it seems like it might be useful as a general reflection of the proportion of under-24 month olds that are being fed according to guidelines. However, the variable is very influenced by age, as illustrated in the proportion of infants/young children that adhere to it in Table 1: there is a steep rise from 6 months onward that corresponds to the inclusion of complementary foods in its makeup. For this reason, it seems to me that a presentation of this variable disaggregated by age category would be more useful, you may find that different factors are associated with it for different age groups.

We agree that this is a valuable approach and have undertaken sub-group analysis by age accordingly. This is described in the Methods (lines 238-241), with a summary in the results (lines 341-357) and the complete results included in new Supplementary Table 1 (Chi-square analyses) and new Supplementary Table 2 (multivariable logistic regression analyses). The subgroup analysis did not change the findings for the antenatal and perinatal service delivery associations with age-appropriate IYCF-recommended breastfeeding practice.

[Methods] “Since the definition of IYCF-recommended breastfeeding practice changes at 6 months of age, we subsequently applied the same analytical approach to two sub-groups based on age: 0-5 completed months (exclusive breastfeeding) and 6-23 completed months (continued breastfeeding with complementary foods).”

I am curious why you elected to treat ANC receipt as a dichotomous variable.

This was due to the original WHO recommendation of a minimum of 4 antenatal visits (0-3; 4+). We have now disaggregated this variable into 0-3, 4, 5-7, 8+ to provide more granular information, and to reflect the new WHO (2016) recommendation of 8+ antenatal visits (see lines 193-195 and Table 1). The new coding did not change the relationship of number of antenatal visits with either outcome.

“Variables related to antenatal and perinatal service delivery were informed by the WHO breastfeeding practice guidelines (21) and the WHO recommendations on antenatal care for a positive pregnancy experience (23): number of antenatal visits (0-3 visits; 4 visits, 5-7 visits, 8+visits)…”

Line 230.. and elsewhere isn’t it age appropriate “feeding” not “breastfeeding” given that complementary foods?

We are focussed on the breastfeeding component and defined age-appropriate IYCF breastfeeding practice from 6-23 months as continued breastfeeding with complementary foods. We did not evaluate the quality or quantity of complementary foods in this study, so did not wish to refer to age-appropriate ‘feeding’.

Table 2. I assume there are typos in the OR’s for age in months here for older age groups.

No – these are correct. We think the reviewer is referring to Age of mother (40-49 years). In this age-group the numbers are very small and hence the 95% confidence interval of the odds ratio is very wide.

Table 2. Is there a reason to not include the unadjusted OR’s for the age-appropriate feeding practices here? (they are blank)

Not sure what the reviewer means here – have carefully checked Table 2 as submitted. Both unadjusted and adjusted ORs are blank for variables that were not included in the models.

Given that significance testing was used to determine which variables were included in final models, when reflecting on these findings vs. previous studies I’d suggest also looking at the unadjusted coefficients.. because the relative strength of associations and other variables that are included in your models are influencing what gets selected.

We have explained our approach to variable selection and confounding in the paper. In our view it would not be helpful to include estimated coefficients for non-significant variables. The unadjusted ORs for all variables with p>0.1 in Chi-square analysis are included in Table 2 and supplementary Table 2.
