# Supplementary material for: Antenatal and perinatal service delivery associations with breastfeeding outcomes in Nepal: Analysis of the 2016 Nepal Demographic and Health Survey
Source: PLOS Glob Public Health. 2023 Apr 17;3(4):e0001824. doi: 10.1371/journal.pgph.0001824 (PMC10109470; doi:10.1371/journal.pgph.0001824)
Supplement: Attachment — Submitted filename: Response to reviewers comments.docx [file pgph.0001824.s004.docx]

**Response to Reviewer 3 (Second review – please see red text)**

Thank you for the opportunity to address the remaining comments of Reviewer 3. This feedback is very valuable, and we have incorporated all the requested changes.

**Reviewer #3:** I'm pasting my responses to the issues that i still have questions about below each of the previous responses beginning with OK (i wish we'd numbered them)

I am curious why you elected to treat ANC receipt as a dichotomous variable.

Author (first review): This was due to the original WHO recommendation of a minimum of 4 antenatal visits (0-3; 4+). We have now disaggregated this variable into 0-3, 4, 5-7, 8+ to provide more granular information, and to reflect the new WHO (2016) recommendation of 8+ antenatal visits (see lines 193-195 and Table 1). The new coding did not change the relationship of number of antenatal visits with either outcome. “Variables related to antenatal and perinatal service delivery were informed by the WHO breastfeeding practice guidelines (21) and the WHO recommendations on antenatal care for a positive pregnancy experience (23): number of antenatal visits (0-3 visits; 4 visits, 5-7 visits, 8+visits)…”

OK- at the time of this survey (2016) Nepal was recommending (and recording) 4 visits on ANC cards, this is changing. I do think there is value in assessing a finer stratification for those with <4 visits rather than lumping 0-3 together.. as 0 visits suggests non use of health services completely which is very different than seeking some care.. and 4 was “complete” adherence to recommendations at the time.

Author (second review): We have disaggregated this variable into 0, 1-3, 4, 5-7, 8+ antenatal visits as per the reviewer’s request. Changes have been made in the text (line 170 'Revised Manuscript with Track Changes') and in Table 1. After this reclassification the associations (Chi-square) between the number of ANC visits and ‘breastfeeding within one hour of delivery’ and ‘age-appropriate feeding practice’ remain not significant (p=0.189 and p=0.159 respectively).

We have also included an additional phrase and sentence in the Discussion regarding antenatal visits (lines 362-367 'Revised Manuscript with Track Changes').

“The lack of association between antenatal or perinatal service delivery and ~~age-appropriate breastfeeding practice~~ exclusive breastfeeding to 6 months of age in our study suggests a need to include information about exclusive breastfeeding in these services and to provide additional postnatal support ~~may be necessary to increase the rate of exclusive breastfeeding to 6 months~~. Another study found very few mothers received any information on breastfeeding during antenatal visits in Nepal (27).”

Line 230.. and elsewhere isn’t it age appropriate “feeding” not “breastfeeding” given that complementary foods?

Author (first review): We are focussed on the breastfeeding component and defined age-appropriate IYCF breastfeeding practice from 6-23 months as continued breastfeeding with complementary foods. We did not evaluate the quality or quantity of complementary foods in this study, so did not wish to refer to age-appropriate ‘feeding’.

OK- I find this confusing.. if complementary foods are included in the indicator, it’s more than just breastfeeding.

Author (second review): We have changed the terminology throughout the document to ‘age-appropriate feeding practice’ or ‘age-appropriate infant and young child feeding (IYCF) practice’.

Table 2. I assume there are typos in the OR’s for age in months here for older age groups.

Author (first review): No – these are correct. We think the reviewer is referring to Age of mother (40-49 years). In this age-group the numbers are very small and hence the 95% confidence interval of the odds ratio is very wide.

OK What I am looking at are the unadjusted OR’s and the corresponding 95% CI’s for age groups 12-17 months and 18-23 months. The 95% CI’s do not include the OR coefficients within their bounds.

Author (second review): The reviewer has correctly identified typos on these ORs – thank you. Table 2 has been corrected.

Table 2. Is there a reason to not include the unadjusted OR’s for the age-appropriate feeding practices here?(they are blank)

Author (first review): Not sure what the reviewer means here – have carefully checked Table 2 as submitted. Both unadjusted and adjusted ORs are blank for variables that were not included in the models.

OK What I am suggesting is rather than leaving blank space for something that was actually tested, that you include the unadjusted OR’s in that space. This also would enable comparison vs.previous studies (as mentioned below).

Author (second review): We have now included the unadjusted ORs for all variables tested in Table 2.
